# The epigenetic landscape in intestinal stem cells and its deregulation in colorectal cancer

**DOI:** 10.1093/stmcls/sxae027

**Published:** 2024-04-10

**Authors:** Axelle E M Larue, Yaser Atlasi

**Affiliations:** Patrick G Johnston Centre for Cancer Research, Queen’s University Belfast, Belfast BT9 7AE, United Kingdom; Patrick G Johnston Centre for Cancer Research, Queen’s University Belfast, Belfast BT9 7AE, United Kingdom

**Keywords:** epigenetics, chromatin, intestinal stem cells, colorectal cancer

## Abstract

Epigenetic mechanisms play a pivotal role in controlling gene expression and cellular plasticity in both normal physiology and pathophysiological conditions. These mechanisms are particularly important in the regulation of stem cell self-renewal and differentiation, both in embryonic development and within adult tissues. A prime example of this finely tuned epigenetic control is observed in the gastrointestinal lining, where the small intestine undergoes renewal approximately every 3-5 days. How various epigenetic mechanisms modulate chromatin functions in intestinal stem cells (ISCs) is currently an active area of research. In this review, we discuss the main epigenetic mechanisms that control ISC differentiation under normal homeostasis. Furthermore, we explore the dysregulation of these mechanisms in the context of colorectal cancer (CRC) development. By outlining the main epigenetic mechanisms contributing to CRC, we highlight the recent therapeutics development and future directions for colorectal cancer research.

## Graphical Abstract

**Graphical Abstract:**
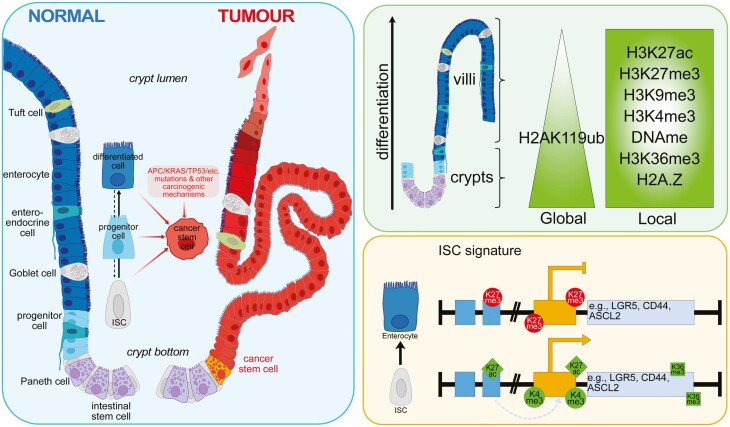

## Introduction

Epigenetic mechanisms control gene expression without altering the DNA code, ultimately regulating cell plasticity in both normal homeostasis and pathophysiological conditions. A prime example of epigenetic regulation occurs during stem cell differentiation, where cells change their identity without altering their genetic code.^1^ Here, stem cells constantly navigate the delicate balance between self-renewal and lineage differentiation. This process is controlled by specific epigenetic mechanisms that can silence self-renewal genes and activate differentiation genes during stem cell differentiation. Lineage differentiation typically follows a predetermined path in which cells become “locked” into their designated cell fates, preventing them from de-differentiating under normal physiological conditions. In addition, cellular de-differentiation can also occur during tissue repair and wound healing allowing tissue regeneration.^2^ Furthermore, the deregulation of epigenetic mechanisms can lead to disease states such as cancer by altering or blocking normal cell differentiation or inducing cellular de-differentiation. It is important to note that there is a close interplay between genetic mutations and epigenetic deregulation in cancer. For example, many key chromatin regulators are often mutated in tumors, leading to epigenetic deregulation and uncontrolled gene expression.

In this review, we utilize the intestine as a prototype example of adult tissues that undergo continuous self-renewal and regeneration. We explore the epigenetic landscape of intestinal cells, from intestinal stem cells (ISCs) to differentiated progenies (eg, enterocytes), and we address how the intestinal epigenome is deregulated in colorectal cancer (CRC).

## Main

### Mechanisms of chromatin activation and repression during intestinal stem cell differentiation

**Figure 1.**
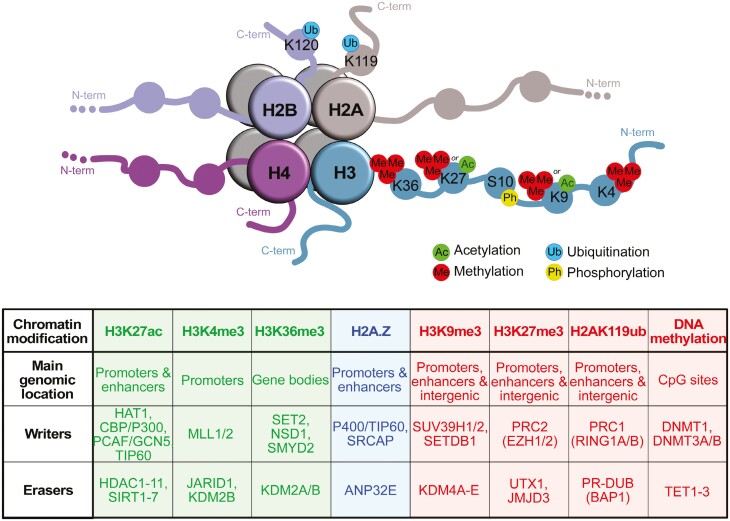
Schematic representation of different histone modifications discussed in this review. Histone tails can be chemically modified at specific residues (eg, lysine 9 and 27). Table summarizing the chromatin modifications associated with different residues, their genomic localisations, functions (green = activating mechanism, red = repressive mechanism, blue = both), and examples of associated epigenetic writers and erasers.

**Figure 2.**
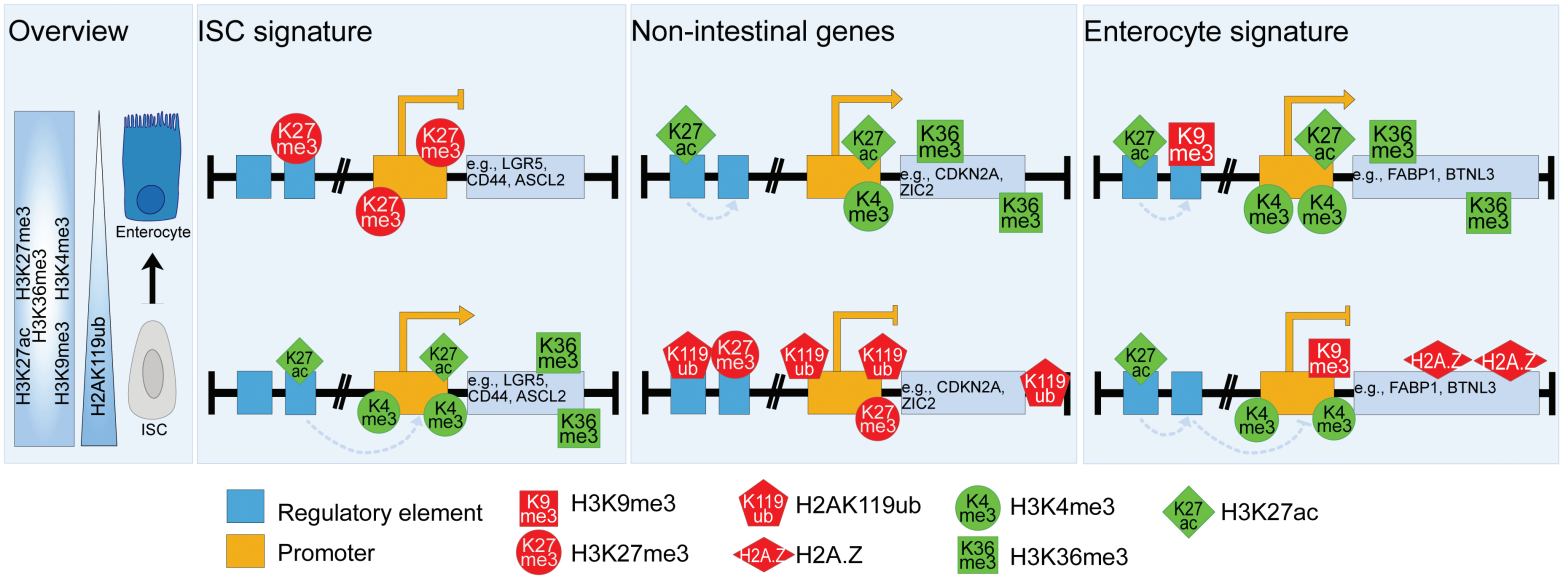
Patterns of epigenetic remodeling in intestinal crypt-villus differentiation. During stem cell differentiation, the ISC signature is silenced by replacing active marks H3K27ac and H3K4me3 with H3K27me3 at loci including Lgr5, Cd44, and Ascl2. In contrast, the enterocyte signature genes (eg, Fabp1, *Btnl3*) are activated by the loss of H3K9me3 mark at promoter regions and gain of H3K27ac at loci already marked with H3K4me3. Important lineage differentiation genes (eg, Zic) and *Cdkn2a* are repressed by H2AK119ub1 and H3K27me3 in ISCs, ensuring ISC self-renewal.

Various epigenetic modifications are involved in regulating the transition from stem cells to differentiated cells in intestine^3^ (Figure 1). Notably, these epigenetic mechanisms operate rapidly in the gut, where intestinal stem cells (ISCs) can differentiate into enterocytes within 3–5 days. Emerging evidence highlights that ISCs, and differentiated cells share some epigenetic features, potentially contributing to cellular plasticity used in intestinal tissue repair.^4-8^ Here, we explore the functions of various epigenetic marks through the formation of intestinal crypts-villi axis, as summarized in Figure 2.

#### H3K27me3 maintains the correct balance between absorptive and secretive lineages

The overall deposition of H3K27me3 at promoters and gene bodies is similar between crypt and villi cells. Nevertheless, there are important genes associated with crypt differentiation (eg, Lgr5, Ascl2) that exhibit differential deposited H3K27me3 that is accompanied with differential gene expression.^9,10^ In particular, the ISC marker, *Lgr5*, is part of a set of genes that gained significant H3K27me3 mark in villus cells and whose expression is repressed during the transition from ISCs to differentiated intestinal cells. In line with these findings, analysis of intestinal organoids confirmed a small number of loci (including *Lgr5* and *Sox9*) that gain H3K27me3 mark during stem cell to enterocyte differentiation.^11^ Using a system called DCM-TM (bacterial DCM methylation fused to RNA polymerase 2 subunit b), which can tag transcriptionally active genes at different developmental stages, showed that genes such as *Cd44*, *Zic1*, and *Zic4* gain H3K27me3 at enhancers and promoters during the transition from crypts to villi, although global H3K27me3 levels between the 2 compartments remain similar.^12^ Of note, the changes in H3K27me3 deposition at intergenic regions, which constitute the largest number of H3K27me3 peaks, remain less studied.^9^

The repressive H3K27me3 histone mark is deposited by the Polycomb Repressive Complex PRC2, composed of core *EED*, *SUZ12,* and *EZH1/2* (catalytic activity) proteins. *Eed* deletion in crypts leads to a reduced proliferation rate, followed by crypt shrinkage, and stunted and dysmorphic villi.^9,13^ A suggested mechanism for this phenotype is the upregulation of *Cdkn2a*, which is normally repressed by PRC2, leading to slower cell cycle progression observed in the *Eed*^−/−^ crypts. However, this phenotype is not sufficient to compromise the overall intestine homeostasis. Additionally, in vivo deletion of PRC2 components (*eg, Ezh1/2*, *Eed*, and *Suz12*) does not overtly activate gene expression, and transcriptional activation was only observed at bivalent sites demarked by both H3K27me3 and H3K4me3.^9,14^ Interestingly, although *Eed*-deletion does not impact ISCs, it increases the number of goblet cells and triggers an aberrant lineage commitment toward the secretory path.^15,16^ Furthermore, although *Eed* deletion does not compromise ISC maintenance, it strongly impairs cryptcell regeneration upon irradiation, and ISCs exhibit reduced capacity for organoid formation ex vivo.^15^ Notably, depletion of H3K27me3 in the intestine does not necessarily affect the deposition of PRC1 repressive mark H2AK119ub, demonstrating that PRC1 and PRC2 activities may have some uncoupled functions in intestinal (stem) cells.^15,17,18^

#### H2AK119ub represses non-intestinal genes in ISCs

Until recently, the role of the PRC1-deposited H2AK119ub repressive histone mark remained elusive in intestine. Emerging data suggest that H2AK119ub preserves the stemness in intestine independently of H3K27me3.^17^ H2AK119ub is highly deposited in ISCs and is strongly reduced during intestinal stem cell differentiation.^18^ Staining of RING1B and the non-canonical PRC1 protein CBX3 also revealed high expression in ISCs.^12^

Deletion of catalytic PRC1 core subunits *Ring1a/b* (and consequent loss of H2AK119ub) in mice leads to rapid defects in the gut lining, and compromises crypt homeostasis. Loss of PRC1 also reduces the number of ISCs, and results in abnormal and cystic-like crypts, suggesting a functional role of PRC1/H2AK119ub in ISC renewal and maintenance.^18^ Here, deletion of *Ring1a/b* and subsequent H2AK119ub depletion results in induction of multiple transcription factors, including the ZIC proteins that normally interact with and inhibit the TCF/ β-catenin binding to chromatin.^19^ Additionally, the majority of genes marked by H2AK119ub in ISCs do not belong to the intestinal lineage (eg, ZIC family). These data suggest that PRC1/H2AK119ub contributes to preserving the stem cell identity via repressing TFs associated with gut differentiation pathways (such as *Zic2*).^12,18,20,21^

#### H3K9me3 and heterochromatin in ISCs

The H3K9me3 mark is deposited by the methyltransferases SUV39H1, SUV39H2, and SETDB1,^22^ and serves as a binding site for recruiting constitutive heterochromatin proteins such as heterochromatin protein 1 (HP1).^23^ Although studies on H3K9me3 mark in ISCs and gut cells are limited, it was demonstrated that crypt cells are moderately enriched with H3K9me3 compared to villi cells. H3K9me3 deposition between crypt and villi cells at promoters has been described as “static.” However, enhancers near *Cd44*, the histone-lysine N-methyltransferase *Smyd1*, and the enterocyte gene *Fabp1* gain H3K9me3 in villi when compared to crypt cells.^12^

Deletion of methyltransferase *Setdb1* in mouse organoids and spheroids leads to significant loss of H3K9me3 deposition. This results in excessive stem cell death, and altered development of intestinal crypts which exhibit an ulcerative, ischemic, and edemic phenotype.^24^ Importantly, mouse bowel inflammation was triggered by the knockout of *Setdb1*, accommodated by lymphocytes penetration into the lamina propria and epithelium. This phenotype is partially attributed to the activation of ERVs (endogenous retrovirus-like elements with long repeats) that mimic viral infection via production of double-stranded RNAs. In return*, Z-DNA binding protein 1* (*ZBP1*) becomes activated and mediates the inflammatory or necroptotic death of ISCs. As a result, epithelial homeostasis is lost, leading to crypts degeneration and villi atrophy. In drosophila, age-dependent downregulation of H3K9me3 in enterocytes induced oxidative stress resulting in genomic stress and apoptotic in ISC. This highlights a potential passive regulatory role of H3K9me3/HP1 in ISC homeostasis.^25^

#### DNA methylation

Whole-genome analysis of DNA methylation in *Lgr5*-expressing stem cells and differentiated progenies, revealed that, globally, DNA methylation exhibits a stable pattern across the genome.^5,26^ However, there are significant changes in DNA methylation at regulatory regions, and particularly at enhancers. A larger number of regulatory elements that gain DNA methylation during ISC differentiation, are located at sites associated with ISC biology including genes involved in WNT-signaling. In contrast, regions that lose DNA methylation are largely associated with genes involved in enterocyte metabolism, and concomitantly, gain H3K27ac during ISC differentiation.^26^ In line with these findings, in vivo deletion of *Dnmt1* in adult mice triggers tissue expansion via loss of DNA methylation at ISC-specific enhancers.^26^ This dysregulated epigenome is also accompanied by increased proliferation of secretory progenitors with an altered morphology.^27^ Interestingly, *Dnmt3b* knockout has little impact on the intestine,^28^ and it was reported that overexpression of *Dnmt3b* (observed in colorectal tumors) may be more deleterious as it increases the number of adenomas in the colon.^29^

DNA methylation loss may represent the first step to generate the ISCs epigenetic profile during ESCs to ISC differentiation. Comparison between mouse ESCs and adult ISCs, revealed dynamic changes in DNA methylation.^4^ 40% of regions lost 5mC and approximately 20% gained methylation during this transition. The progressive 5mC loss continues further as ISCs differentiate along the colonic crypts. In line with these findings, DNA methylation is lost at ISC signature genes (eg, OLFM4 and *AXIN2*) in ISCs vs ESCs, followed by increased H3K27me3 deposition and the presence of bivalent active marks H3K27ac and H3K4me3 is observed at these sites.^26^ This all-inclusive epigenome, held by DNA methylation, may represent a precursor trait of the intestinal characteristic plasticity, which has been reported to offer cell phenotype switching (eg, from absorptive to secretive lineage).

#### H2A.Z. maintains intestinal homeostasis and facilitates other epigenetic modifications

Three main complexes, p400/Tip60 and SRCAP are able to incorporate histone variant H2A.Z at target regions.^30-33^ H2A.Z is enriched at promoters and enhancers and exerts multiple functions in chromatin regulation, including both transcriptional activation and repression. For example, H2A.Z is deposited at promoters of lineage differentiation genes in ISCs, thereby enabling ISCs to preserve their self-renewing capacity.^4,34^ Accordingly, during ISC-to-enterocytes differentiation, H2A.Z level is globally decreased across promoters resulting in transcriptional activation of many enterocyte-specific genes. Interestingly, a large number of these genes are already primed by H3K27ac and H3K4me3 in ISCs.^4^ Similarly, retrospective analysis of enhancer activity during ISC differentiation, showed that enterocyte-specific enhancers are primed by H2A.Z in ISCs, and are fully activated in enterocytes. Motif analysis of enhancers marked with H2A.Z in enterocytes revealed enrichment of Notch- and EGF-associated TFs, indicating potential roles of these signaling pathways in controlling the enhancer activity in enterocyte signature genes.^12,35^ Thus, it appears that in ISCs, H2A.Z pre-mark promoters and enhancers that are further activated in enterocytes.

#### H3K36me3 contributes to intestinal immunity

Nuclear receptor SET domain-containing 1 (NSD1) and SET domain-containing 2 (SETD2) methyltransferases are the most described proteins able to catalyze the methylation of histone H3 at Lysine residue 36, with SETD2 being specifically required for tri-methylation.^36^ H3K36me3 mostly marks actively transcribed gene bodies.^37^ and is part of the homologous recombination (HR) repair response to DNA damage, and stabilizes the genome by preventing mutations that follow double-strand breaks.^38-40^ In normal physiology, deletion of *Setd2* has no major effect on intestinal homeostasis or ISC lineage differentiation. However, deletion of *Setd2* enhances the self-renewal of intestinal stem cells and tissue regeneration upon tissue injury such as irradiation. Mechanistically, deletion of *Setd2* leads to alternative splicing of genes implicated in tumorigenesis, such as disheveled segment polarity protein 2 *Dvl2*, leading to elevated WNT signaling.^41^ Furthermore, the dynamic balance between methylated and demethylated H3K36me3, maintained by methyltransferases like SETD2 and demethylases from the Jumonji domain (KDM4/JMJD2) family, is crucial for cellular homeostasis (eg, DNA repair), by serving as a binding dock for effector proteins (readers) that recognize histone modifications and mediate cell fate. SETD2 notably can modulate splicing factors to prevent histone exchange over exons. SETD2 suppresses the addition of newly acetylated histones and also relays the deacetylated state of transcribed regions, thereby maintaining RNA polymerase II-coordinated transcription.^40^ The function of SETD2 may become important in tumorigenesis where aberrant expression of tumor suppressor genes is observed.^42^ Additionally, SETD2-mediated H3K36me3 deposition was reported to contribute to the function of group 3 innate lymphoid cells (ILC3s), that are crucial immune cells in the intestinal.^43^ ILC3s lymphocytes patrol the intestinal mucosa and target foreign antigens in the gut lumen, promoting epithelial barrier integrity and intestinal homeostasis. Therefore, SETD2 also indirectly contributes to intestinal homeostasis by influencing intestinal immunity.^44^

#### H3K4me3

H3K4me3 mainly marks promoters and is deposited by the COMPASS-like proteins MLL1/2 and SET1 methyltransferase, which interacts with other core subunits, including WDR5, RBBP5, ASH2L and DPY30.^45^ During the transition between ISCs and enterocytes, H3K4me3 shows little changes on the global levels. However, the ISC signature genes such as *Lgr5* show dynamic H3K4me3 pattern at their promoters and lose the mark during ISC-to-enterocyte transition.^4^ Interestingly, many enterocyte associated genes such as *Txn1* or *Prdm1* are already marked with H3K4me3 in ISCs before their transcription.^4^ Epigenetically, intestinal progenitors share characteristics that are also found in ISCs.^2^ Indeed, similarities between secretive and absorptive gut progenitors suggest a high degree of epigenetic plasticity in the small intestine in which lineage specificity may depend on transcription factors. Of interest, a recent publication on digestive organogenesis in Zebrafish demonstrated that *wdr5* (and H3K4me3) coordinate cell proliferation, differentiation in gut. Genetic deletion of *wdr5* and loss of H3K4me3 results in cells with a progenitor-like status, with high proliferation rate. This defects in differentiation program is attributed to the role of H3K4me3 in promoting differentiation and anti-proliferation genes. Here, *wdr5* and H3K4me3 can promote *apc* expression to reduce WNT-signaling in differentiated cells, and to maintain the expression of differentiation-associated genes (eg, fabp2 and *chia.1*). H3K4me3 was also shown to promote the expression of anti-apoptotic genes which modulate P53 function to ensure differentiated cell survival.^46^

#### H3K27ac marks regulatory elements and supports ISC maintenance

Acetylation of H3 at lysine residue Lys27 serves as a marker of active chromatin. Because this modification is located at Lys27, the H3K27ac, and H3K27me3 marks are mutually exclusive and play functionally distinct roles in chromatin regulation.^47^ Of note, H3K27ac is deposited by the histone acetyltransferases (HATs) including the p300/CBP complex primarily at promoters and enhancers.^47-49^ A larger number of regulatory elements are marked by H3K27ac in ISCs and progenitor cells compared to enterocytes. These dynamic changes between ISCs and differentiated cells are particularly prominent in ISC genes such as *Lgr5* and *Lrig1* and proliferative genes (eg, Sox9, *Myc*) which are highly marked by H3K27ac in ISCs.^4,26^ Consisting with these observations, the HDAC inhibitors valproic acid and trichostatin A, which are known to elevate total protein-acetylation, are used in organoid cultures to expand the stem cell population. This results in organoids enriched with Lgr5-positive cells.^50^ In contrast to ISC vs enterocytes, similar levels of H3K27ac are found in absorptive and secretory progenitor cells, underscoring the shared differentiation pathway between the 2 lineages. This further emphasizes the permissive nature of chromatin in the intestine.^35,51^

The dynamic changes in H3K27ac levels are mediated by a large family of HATs and histone deacetylases (HDACs).^52,53^ Loss of *p300* reduces cell proliferation and the number of cells in the crypt. Importantly, *Lgr5* expression is significantly reduced in *p300*-mutant compared to WT mice.^54^ Deleting *Creb* in mice reduces cell proliferation without affecting gut homeostasis. *Creb*-KO or *p300*-mutant mice exhibit a higher number of goblet cells, suggesting a role for this chromatin regulator in lineage differentiation. In vitro studies also showed that *Creb*-KO ISCs are unable to form organoids, however, this seems to be compensated for in vivo. Nevertheless, the response of these mutant mice to environmental stresses remains to be examined.

#### Long non-coding RNAs

Another class of epigenetic regulators is non-coding RNAs. These transcripts, unable to produce proteins, are divided into 2 main categories based on their length; small noncoding RNAs that are less than 200 nucleotides (*eg*, microRNAs (miRNAs) or Piwi-interacting RNAs (piRNAs)) and long noncoding RNAs (lncRNAs) that are more than 200 nucleotides long.^55^ lncRNAs play various roles in intestinal development, through functions such as modulating the stability and translation of mRNAs (eg, lncRNA *H19*), or functioning as decoys for transcription factors (eg, GAS5). These versatile functions of lncRNAs regulate various aspects of gut physiology, including the critical maintenance of the epithelial barrier, pivotal for ensuring gut homeostasis. Here, lncRNAs influence cell adhesion, migration, and the integrity of tight junctions.^56^ lncRNAs can also influence gene transcription impacting gut renewal, immune modulation, and cytokine production. For example, upregulation of *H19* lncRNA prevents *miR34a* and *let-7* from inhibiting intestinal epithelial cell proliferation and regeneration.^57^ Similarly, *lncRNA-uc.173* can interact with and inhibit *miR195*, promoting renewal of the intestinal epithelium, and contributing to the regeneration of the gut mucosa upon injury.^58^ lncRNAs can also function as epigenetic regulators participating in chromatin remodeling by recruiting chromatin-modifying enzymes (eg, DNMTs, HATs, or HDACs) to specific loci thereby activating or repressing gene expression during intestinal differentiation.^59,60^ For example, *lncRNA-LALC* recruits PRC2 to CpG islands near the promoter of target genes. The induced chromatin changes facilitate DNMTs recruitment and result in the epigenetic silencing of *LZTS1*, a gene known to inhibit cell growth by overexpressing *CDK1*.^61,62^

#### Others

Our comprehension of the histone code is constantly expanding, revealing intricate functional roles for diverse chemical modifications of amino acids within histones’ globular regions or tails. These modifications encompass, amongst others, methylation of arginine residues, ubiquitination of lysines, and phosphorylation. For example, phosphorylated histone marks (eg, phosphorylation of H3 at serine-10 H3S10ph, at serine-28 H3S28ph, or at tyrosine-41 H3Y41ph) can alter the chromatin structure by adding a negative charge to the histone tail, and can be mediated by kinases, including Aurora B or JAK2. One such example is H3S10 phosphorylation, which participates in chromosomal compaction during mitosis. Its deposition increases in the prophase and metaphase stages of cell division, representing an informative mitotic marker. Here, H3S10ph plays a role in chromosome segregation and kinetochore function.^63^ H3S10ph dephospho mutant (serine to alanine substitution) is unable to correctly segregate chromosomes during mitosis, leading to heterochromatin-associated H3K9me2 mark to spread into nearby euchromatin.^64^ H3S10ph also contributes to chromatin de-condensation and transcriptional activation during interphase in response to extracellular signals. Thus, H3S10ph is a dynamic histone mark contributing to both chromatin compaction and de-compaction in response to various stimuli. However, the role of many histone marks remains less studied in ISCs.

### Deregulation of epigenetic marks in colorectal cancer

Maintenance of permissive chromatin in crypt development allows fast crypt recovery and stem cell repopulation upon tissue injury but may also facilitate carcinogenesis. Accordingly, cancer stem cells (CSCs) and ISCs share great similarities, including the ability to give rise to multiple cell lineages, express similar stem markers and rely on WNT-signaling for proliferation.^65^ Similarly, ISCs and CSCs express *CD133*, *CD44*, and *LGR5* among other markers, suggesting a relative resemblance between normal and cancer SCs.^66^ However, they express differences in the regulation of these mechanisms. For example, the induction of the WNT-pathway enables the enrichment of organoids with stem cells,^50^ while WNT-signaling control is lost in CRC CSCs and its hyperactivation may cause aberrant crypt proliferation.^67^ Importantly, aggressive CRC phenotypes were shown to upregulate the ISC transcription signature, marked with overexpressed *Lgr5* and *Ascl2* and lowered *Krt20* expression.^68^ Unlike differentiated cells, CSCs may evade chemotherapy, giving rise to different resistant tumor populations. In this section, we explore how certain epigenetic mechanisms discussed in the previous section may play a role in tumorigenesis, as well as the strategies used to counteract them in cancer therapy.

#### PRC2 and H3K27me3 are associated with CRC tumorigenesis

Although *EZH2* gain-of-function mutations are present in various cancers, notably AML,^69^*EZH2* mutations in CRC are rare and the exact role of PRC2/ H3K27me3 in CRC remains less understood. Nonetheless, most studies demonstrate that activity of PRC2 is associated with CRC tumor proliferation and progression.^70-72^ For example, overexpression of *EED*, *SUZ12,* and *EZH2* is significantly correlated with decreased disease-free survival in CRC,^71^ and targeting EZH2 reduces tumorigenesis by inducing autophagic and apoptotic pathways and G1/S cell cycle arrest.^73^ Knockdown of *EZH2* was also shown to result in reduced WNT/β-catenin signaling, a pathway known to promote CRC tumorigenesis.^74^ H3K27me3 also represses *IHH* (Indian Hedgehog), a gene that normally is associated with colonocyte differentiation.^67,70^ In addition, mouse xenograft models showed that pharmacological inhibition of EZH2 with Tazemetostat (EPZ-6438 compound) significantly reduces tumor volume and size. This is in line with clinical studies which demonstrated that patients with EZH2 + tumors had a poor prognosis and lowered survival rate compared to EZH2-negative CRCs.^75^ A few studies have also reported a potential role of EZH2 in CRC metastasis. For example, in TGF-β-induced epithelial-to-mesenchymal transition (EMT) in SW480 cells, the promoters of *WNT5A* and *CDHA1* genes become occupied with EZH2 and marked with H3K27me3. Furthermore, treatment with EZH2-inhibitor during EMT rescues *WNT5A* and E-cadherin expression and restores cell-adhesion, which is normally lost during TGF-β induced EMT.^76^

In addition to writers of H3K27me3, the erasers of this histone mark also play a role in CRC. The H3K27me3 demethylases KDM6A (UTX1) and KDM6B (JMJD3) are more expressed in crypt cells compared to villi, suggesting a potential role in intestinal stem cells.^77^ However, their roles in carcinogenesis remain contradictory. KDM6A supports tumor-initiating cells and its targeting leads to enhancer reprogramming and substitutivity to chemotherapy. In contrast, *KDM6B* inhibits cell proliferation, induces apoptosis and correlates better with overall survival.^78^ Despite these observations, little is known about H3K27me3 in CSCs, and only a handful of studies investigated how these epigenetic marks influence the response of CRC cells to standard-of-care chemotherapy. For instance, it was shown that the chemotherapeutic drug oxaliplatin results in reduced H3K27me3 in CRC cells due to increased expression of *KDM6A* and *KDM6B*. Interestingly, elevating the level of H3K27me3 in conjugation with oxaliplatin can enhance the efficacy of chemotherapy.^79^ In this regard, KDM inhibitors have recently gained attention for targeting CRC CSCs. KDM6A inhibition using GSK-J4 compound leads to an increase in the global levels of H3K27me3, accompanied by downregulation of H3K27ac at promoter and enhancer regions of genes *ID1* and *TERT*, both identified as novel markers of intestinal stemness.^80^ Thus, *KDM6A* silencing may also lead to enhancer reprogramming that can interfere with the maintenance of CRC CSCs.^77^

#### The involvement of PRC1/H2AK119Ub in CRC

Little is known about the precise role of H2AK119ub in CRC. However, several studies suggest a contribution of the canonical and non-canonical PRC1 components in CRC. The cPRC1 characterized by Polycomb Group Ring Finger 2 and 4 (PCGF2 (MEL18) and PCGF4 (BMI1)) mediates CRC development.^81^ This function of PRC1 can be, in part, attributed to promoting the JAK-STAT3 pathway. For example, *Bmi1*- and *Mel18*-dKO mice have reduced number of polyps, and decreased *Stat3* expression which results in downregulation of STAT3-target genes (eg, cyclins, *Myc*) and upregulation of *p21* and *Bcl10*.^81^ Similarly, knockdown of *BMI1* in CD133+/CD44 + HCT116 cells increases E-cadherin expression and reduces EMT-mediated invasion and migration in these stem-like cancer cell populations.^81,82^

The core component of PRC1 complex also has significant roles in CRC. For instance, RING1B (*RNF2* gene) is upregulated in CRC tissues.^83^ Silencing of *RNF2* in HCT116 cells leads to decreased H2AK119ub levels and, consequently, upregulation of p21 and EGR1 acting as a tumor suppressor, thus contributing to reducing cell proliferation and promoting apoptosis.^84^ RING1A can also contribute to cancer development. Here, RING1A was shown to ubiquitinate p53 and consequently reduces its protein levels, leading to increased cancer cell proliferation and survival.^85^

CBX2/4/8 (components of the cPRC1) also play important roles in CRC. *CBX2* overexpression is associated with poor CRC prognosis and its knockdown leads to apoptosis in HCT116 and HT29 CRC models.^86^ Similarly, *CBX8* knockdown in CRC cell lines reduces cell viability and tumor xenografts in mice, likely via affecting p53-signaling.^87,88^ In contrast to CBX2/8, *CBX4* appears to have a tumor-inhibitory functions as its overexpression in the CRC cell line DLD1 reduces cell migration, while knockdown of *CBX4* in HCT116 cells increases tumor size and metastases. Here, downregulation of *RUNX2*, a pro-metastatic marker in CRC, by the CBX4-HDAC3 complex was proposed as a potential mechanism behind the anti-tumor function of *CBX4* in CRC.^89^

The role of ncPRC1s is less studied in CRC. In CRC spheroid models, silencing of ncPRC1.1 core component *PCGF1* resulted in smaller and slowly-growing tumors in mouse xenografts.^90^ Intriguingly, little change in H2AK119ub level was observed upon knockdown of *PCGF1* suggesting that this ncPRC1’s functions may be independent of H2AK119Ub regulation. Accordingly, knockdown of *PCGF1* led to global reduction in active mark H3K4me3 and increased repressive histone mark H3K27me3 at promoter regions of stem markers *CD133* and *CD44*.

CBX3, another component of ncPRC1 is upregulated in CRC,^91^ and promotes cell cycle progression by inhibiting p21 and CDK6, thus supporting metastasis, lymph invasion, and tumor progression.^92,93^*CBX3* was recently identified as a transcriptional regulator of potential biomarker NCAPG, whose levels are also upregulated in CRC. Overexpression of the CBX3-NCAPG axis activates WNT-signaling, known to mediate CRC progression, notably by promoting EMT and suppressing apoptosis in cancer cells.^94^ Significantly, high *CBX3* transcriptional activity was associated with poor disease-free survival in CRC patients, while *CBX3* expression correlates with immune cell infiltration (macrophages and CD8 + T cells) in colorectal tumors.^95,96^ This indicates that *CBX3* may act as a CRC oncogene and participates in CRC progression. Clinically, *CBX3* may be used as a potential prognostic marker in the future.

#### H3K9me3 repression of non-coding regions is critical in CRC

The role of H3K9me3 in CRC is intriguing as its contribution in multiple mechanisms of cancer development and progression. Because of its role in silencing transposable elements (TEs), dysregulation of H3K9me3 may cause strong DNA damage and re-activation of silenced genes.^97,98^ Of note, upregulation of H3K9me3 is observed at the invasive front of CRC tumors suggesting a contribution to tumor invasion and cell motility.^99^ Knockdown of *SETDB1* in SW480 and HCT116 CRC cell lines, reduces H3K9me3 and inhibits cell proliferation (via induction of *CDKN1A*) and colony formation in vitro and reduces tumor formation in mouse xenografts.^100^

*TIP60* has also been identified as a critical tumor suppressor.^101^*TIP60* prevents the activation of endogenous retroviral elements that cause an inflammatory response.^102^ Indeed, depletion of *TIP60* downregulates H3K9me3 methyltransferases *SUV39H1* and *SETDB1* expression, leading to loss of H3K9me3 at retrotransposon regions. Lastly, *SETDB1* upregulation has been proposed as a mechanism of chemotherapy resistance in multiple cancers, including CRC.^98^ Treatment may induce cell stress through LINE-1 expression, consequently activating the IFN inflammatory response, which is followed by dedifferentiation and epigenetic switch to a resistant phenotype.

These recent findings highlight the potential of using the SETDB1-H3K9me3-p21 axis as a target for CRC therapy. Current pharmacological research has developed multiple SETBD1 inhibitors, however none have reached clinical trials for CRC yet. Zhao et al have nicely summarized the available SETDB1 inhibitors but highlight the risks of activating TEs and inducing genomic instability upon H3K9me3 depletion.^103^

#### Aberrant methylation of tumor suppressor genes promoters in CRC

Aberrant patterns of DNA methylation are characteristic of CRC. The tumor genome undergoes global hypomethylation that facilitates the transcription of proliferative genes, transposable elements (eg, LINEs) and promoting genomic instability and carcinogenesis. However, promoters of tumor suppressor genes and CpG islands sites tend to be hypermethylated.^104^ This tendency is observed at multiple genes including, *APC, IGF2, EYA4, TIMP3, CDH1, TWIST1, MLH1*, etc.^105,106^ Transcriptional silencing of *MLH1* gene via promoter hypermethylation, observed in 10%-20% of CRC tumors, leads to genomic instability.^107^ As part of the DNA mismatch repair system, MLH1 contributes to DNA damage signaling and when along other proteins, may provide a single-strand DNA break near the mismatch locus, allowing exonuclease recruitment. Its loss thus participates in microsatellite instability.^108,109^ Similarly, the tumor suppressor *FOXD3* is repressed in CRC by promoter is hypermethylated, leading to activation of the Tuft cell-associated gene, *DCLK1*, in CRC CSCs.^110,111^ Similarly, E-cadherin gene *CDH1* promoter is methylated in CRC, permitting Vimentin and N-cadherin upregulation that leads to a more mesenchymal phenotype, and EMT in CRC.^112^ Of note, inhibiting promoter methylation of anti-tumor genes should promote cancer cell differentiation.^113^ For example, HCT116-enriched CSCs express low *RARB*, whose promoter is methylated, thus contributing to increased radiotherapy resistance. Pharmacological rescue of *RARB* expression using *DNMT1* inhibition improves CSCs’ radiotherapy response.^114^

DNA methyltransferases are rarely mutated in colorectal cancer,^115^ whereas DNA demethylases Ten-Eleven Translocation or TET enzymes can harbor cancer-specific mutations which are found in tumors with elevated DNA methylation. For example, *TET3* loss of function via somatic frameshift mutations are present in up to 30% of CRCs, where *TET3* downregulation may enhance CRC progression.^116^*TET2* is often mutated in leukaemia,^117^ whereas its role in CRC remains less studied. TET2 staining of CRC tissues revealed that *TET2* nuclear expression is lost in aggressive and metastatic tumors.^118^ Similarly, recent findings have highlighted the putative role of *TET2* inactivation in regulating CRC stemness via upregulating TF *ASCL2* expression.^119^

#### H3K27ac deposition induces enhancer reprogramming in CRC

**Figure 3.**
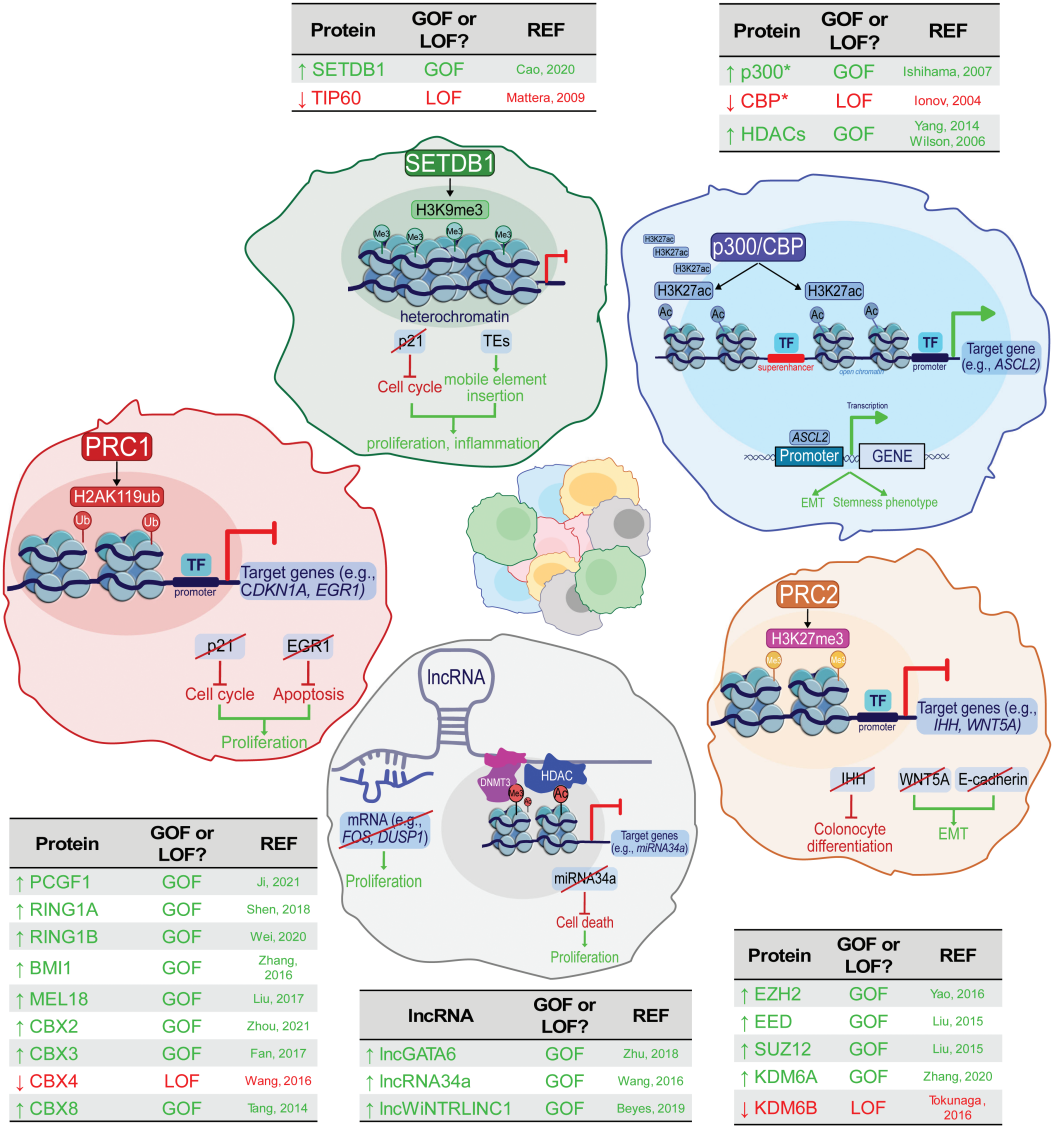
Representative examples of key epigenetic mechanisms affecting CRC development. SETDB1 (top) deposits the heterochromatin mark H3K9me3 to repress transposable elements and *CDKN1A* gene. Thus, targeting SETDB1 represents a strategy to de-repress TEs and suppress cell proliferation. *P300* upregulation in CRC (top right) causes a gain of H3K27ac, affecting genes associated with stem cell identity (eg, ASCL2). Genes inhibiting cell proliferation are repressed by PRC1-deposited H2AK119ub mark (left). Thus, PRC1 overexpression in CRC supports tumor growth. Similarly, PRC2 overexpression (right) represses genes involved in cell differentiation and epithelial markers, further promoting EMT. LncRNAs (bottom) participate in CRC carcinogenesis by promoting proliferation via epigenetic regulation (DNA methylation or miRNA recruitment) or direct gene mRNA interactions.

*P300* and *CBP* are frequently mutated in CRC and in more than 85% of MSI + CRC cell lines.^120^ High expression of *P300*^121^ and low expression of *CBP* are associated with poor prognosis and decreased survival in CRC.^122,123^ While 5-FU-treated tumors show decreased levels of H3K27ac that is associated with degradation of P300/CBP complexes, there is a lack of consensus whether *P300* and *CBP* expressions protect or contribute to CRC progression.^122^ Interestingly, HDAC levels remained unchanged after 5-FU treatment. Similarly, HDAC1, HDAC2, HDAC3, HDAC7, and HDAC8 protein levels are globally upregulated in CRC and characterize a poor tumor prognosis.^124-126^ Silencing or inhibition of these HDACs result in decreased growth and proliferation and increased apoptosis,^125,127,128^ which has led to the development of numerous HDAC inhibitors, currently under preclinical study or early clinical trials in metastatic CRC. A recent study comparing various histone mark patterns in normal gut tissue, tumor samples, and CRC cell lines, demonstrated that H3K27ac at enhancers can distinguish between different stages of CRC progression. Remarkably, when applied to tumor samples, the H3K27ac profiles at enhancers were able to classify the samples to the previously defined consensus molecular subtypes (CMS), demonstrating the importance of H3K27ac histone mark deposition in CRC progression.^129^ In particular, the CMS2 subgroup exhibits a strong correlation between upregulated H3K27ac deposition, de novo enhancer activity and tumor development.^130^ Aberrant H3K27 acetylation notably highlights the role of cancer-specific superenhancers in malignant proliferation.^129^ Aberrant enhancer activity at non-coding regions also participates in CRC tumorigenesis by driving oncogenes expression.^131^ For example, a superhancer near the *ASCL2* transcription factor contributes to the formation of stem-like cancer cells and to the maintenance of intestinal stemness or self-renewal of gut progenitor cells^129,132^ (Figure 3).

Aberrant enhancer activity plays a key role in tumorigenesis. Recent genome wide enhancer profiling using H3K27ac and H3K4me1 ChIP-seq in a large panel of tumor and adjacent normal tissues or in normal crypt versus primary cultures of CRC tumors revealed higher number of gain vs lost enhancers in tumor when compared to normal samples.^131-133^ These gained or lost variant enhancer loci (VELs) correlate well with aberrant gene expression in CRC, and are enriched in CRC risk variants, suggesting that these regions are involved in CRC pathogenesis. Some of these hotspots of aberrant enhancer activity were further validated by CRISPR-interference, showing reduction in cell migration in multiple CRC cell lines, as well as reduced tumor growth for some of the tested super-enhancers.^132^

#### H3K36me3 modulates tumor aggressiveness

*SETD2* is frequently mutated or deleted in human tumors, and aggressive CRC tumors are associated with SETD2 deficiency.^41,134^ By controlling H3K36me3 deposition at gene bodies, SETD2 plays a role in alternative splicing of genes implicated in tumorigenesis. For example, deletion of *Setd2* reduces H3K36me3 deposition, leading to alternative splicing in disheveled segment polarity protein 2 (DVL2) mRNA, and elevated WNT/β-catenin signaling.^41^ Accordingly, *Setd2* deletion stimulates the stemness program and increases the number of tumors in *Apc*-mutant mice. Furthermore, H3K36 trimethylation mediated by SETD2 was shown to play an important role in Treg stability in response to inflammatory intestinal immunity, where tumoral regulatory T cells express elevated SETD2 levels compared to those of adjacent and non-malignant adjacent tissues.^135^ Thus, the SETD2-H3K36me3 axis may offer a potential epigenetic target for CRC therapy.^136^

#### Long non-coding RNAs mediate CRC progression

LncRNAs play a significant role in the development of CRC, notably via epigenetic regulation. These molecules contribute to CRC pathogenesis by influencing gene expression, cell proliferation, metastasis, and chemoresistance. Some lncRNAs may also act as competing endogenous RNAs (ceRNAs) or “sponges” binding to microRNAs and preventing their repressive roles, leading to potential upregulation of oncogenes or downregulation of tumor suppressors, and globally enhancing CRC development.^55^ The lncRNAs Colon Cancer-Associated Transcript-1 and -2 (*CCAT1* and *CCAT2*) have been identified to drive CRC pathogenesis and progression. For example, *CCAT2* is expressed from a region overlapping *c-MYC* super-enhancer. *CCAT2* directly interacts with TCF7L2 (TCF4, the downstream effector of WNT-signaling) stabilizing its binding to *c-MYC* super-enhancer, leading to increased *c-MYC* expression and elevated WNT-signaling. Furthermore, *CCAT2* is also a downstream target of WNT-signaling, thus creating a feedback loop between that promotes metastasis and carcinogenesis.^137^ Similarly, *CCAT1* is also overlapping a *c-MYC* enhancer, and can lead to elevated *c-MYC* transcription.^138^*CCAT1/2* expressions are significantly upregulated in CRC tissues, and their levels correlate with tumor stage, metastasis, and patient prognosis. Consequently, *CCATs* emerge as potential biomarkers for CRC diagnosis, prognosis, and as targets for therapeutic intervention.

Other examples of lncRNAs involved in CRC include *lncGATA6*, which is highly expressed in ISCs and promotes *LGR5* expression to maintain stemness. Importantly, *lncGATA6* was found upregulated in CRC cancer stem cells, and its knockout or inhibition using antisense oligonucleotides, resulted in reduced *LGR5* + cells, tumor size and better overall survival in mice.^139^Similarly, TF and ISC marker *ASCL2* expression can be modulated in CRC by upstream lncRNA *WiNTRLINC1* which is induced by WNT-signaling. This collaboration between the lncRNA and the TCF/β-catenin complex creates a positive feedback loop further promoting *ASCL2* expression.^140^ Another lncRNA, *lncRNA34a*, may induce DNA methylation via recruitment of DNMT3a and HDAC1 to the promoter site of *miRNA34a*, a downstream target of p53, able to silence proliferative genes in stem cells.^141^ Because increased levels of *lncRNA34a* have been observed in colorectal cancer stem cells, this *miRNA34a* silencing mechanism highlights the roles of lncRNAs in of CRC cell proliferation.^142^

lncRNA modifications can also control their functions. For example, the N6 methyladenosine (m6A) methylation modification that is deposited on mRNAs and lncRNAs, can influence RNA degradation or translation.^143^ m6A RNA modification was recently profiled in 5FU-resistant HCT15 cells, revealing that the m6A methylation of lncRNAs may regulate mRNA expression of drug resistance-associated genes, promoting cancer progression. Although the mechanism behind this association remains unclear, the silencing of 2 identified lncRNAs associated with mRNAs of *FOS* and *DUSP1* genes, showed enhanced proliferation and metastasis.^144^ These examples and many others illustrate the importance of lncRNAs in signaling pathways that affect gut homeostasis (reviewed in detail for example in^145-149^).

### The crosstalk of histone modifications in ISCs and CSCs

The interplay between diverse epigenetic modifications underlie chromatin regulation. This is particularly relevant at histone marks where multiple competitive modifications can occur. For example, H3 lysine residues K27 and K9 can be acetylated or methylated, procuring either chromatin activation or repression. This topic has been thoroughly explored in prior studies,^150^ and we provide a selection of illustrative examples in this section. One such example is the H3K27me3/H3K4me3 bivalency, which is characterized by the presence of both active and repressive marks at lineage differentiation loci poised for full activation or repression. For example in ISCs, approximately 25% of H3K27me3-marked promoters also carry H3K4me3. The genes associated with bivalent sites are usually lowly or not expressed in ISCs nor in differentiated cells. Instead, bivalency is believed to represent most loci that were transcribed at an earlier stage of development and became silenced in ISCs.^13^ Of note, the loss of PRC2 is not immediately sufficient to remove H3K27me3, and bivalent sites become transcribed after many replications when PRC2 is depleted. This suggests that H3K27me3 deposition is diluted among the parental nucleosomes and H3K27me3 is progressively reduced, allowing the active mark H3K4me3 to slowly re-activate the bivalent regions.^14,151^

Another example is the interplay between H2AK119Ub (PRC1) and H3K27me3 (PRC2) histone modifications. Notably, canonical PRC1 (cPRC1.2/4) can be recruited to sites marked by H3K27me3 which is deposited by PRC2. Here, H3K27me3 is recognized by the PRC1 subunits CBX readers, which in return can deposit the H2AK119Ub, linking PRC2 and PRC1 at the molecular level.^152,153^ For example, genes upregulated as a consequence of PRC1 ablation, including *Zic* genes and WNT associated *Lgr5*, *Axin2* and *Ascl2* genes, were also up- or downregulated in *Eed*-KO mice, suggesting that both PRC1 and PRC2 share ISC target genes.^16,18^ These common targets are important for the development of CRC, where *Zic* gene family is involved in EMT, metastasis, and proliferation,^154^ while the WNT signaling pathway is considered a driver of colon cancer.^155^ However, variant non-canonical PRC1 (vPRC1.1/3/5/6) can also bind the chromatin independent of H3K27me3 and deposit H2AK119Ub mark. In this regard, conditional *Eed*-KO in mice did not affect the H2AK119ub deposition in intestinal cells. Additionally, ablating PRC1 via genetic deletion of *RING1A/B* does not have major effect on deposition of H3K27me3 at the global levels,^18^ indicating that PRC1 and PRC2 may also exert independent functions in ISCs.

The crosstalk between histone methylation and DNA methylation has also been extensively studied. For example, an inverse relationship between DNA methylation and PRC2 binding has been observed at CpG island rich promoters, allowing gene repression by the PRC2 complex. In contrast, DNMT3A can recognize H3K36me3 via its PWWP domain and increase DNA methylation at gene bodies, contributing to gene activation, transcriptional elongation and alternative splicing. In colorectal cancer, promoters of tumor suppressor genes aberrantly gain DNA methylation at CpG islands (eg, at *CDKN2A*, *MLH1 genes*), leading to gene repression. Many of these CpG islands are repressed by PRC2 in normal cells, suggesting that the loss of H3K27me3 at these sites or the altered activity of DNMTs in cancer may lead to aberrant DNA methylation of PRC2 targets.^156-159^ However, the exact mechanisms underlying the gain of DNA methylation at PRC2 target CpG islands in cancer remain not fully understood. DNA methylation also crosstalk with other methylated histone marks, including H3K9me3 and H3K4me3, that has been reviewed in detail.^160^ Furthermore, lncRNAs’ crosstalk with histone modifications is beyond the scope of this review and has been extensively reviewed by others.^55,161^

### Pharmacological targeting of CRC cells

Epigenetic therapy in colorectal cancer represents an active and promising area of research, with several ongoing clinical trials assessing their potential benefits. Epigenetic drugs, including DNMT and HDAC inhibitors, are under investigation in clinical trials for colorectal cancer, aiming to reverse abnormal epigenetic modifications and suppress cancer growth or metastasis. However, still no clinical trials with epigenetic drugs have yet reached phase III in CRC (Table 1, adapted from Luo et al^162^). The low specificity of epigenetic drugs often causes toxicity and strong discomfort in patients and remains the main challenge for future drugs. Indeed, hematologic malignancies seem to respond better to epigenetic targeting, as opposed to solid tumors, revealing another layer of complexity in cancer epigenetics.^163^ Moreover, the pursuit of effective epigenetic therapies for colorectal cancer encounters a significant hurdle when it comes to targeting colorectal CSCs. These CSCs possess remarkable phenotypic and functional plasticity, enabling them to continuously reshape the tumor’s landscape. For example, depleting *LGR5* + expression in human colorectal cancer mouse xenografts using irinotecan, a topoisomerase I inhibitor, causes the neighboring tumor cells to become resistant (LGR5-). Drug-resistant *LGR5*- cells were also able to re-express *LGR5* days after treatment^164^ and become CSC-like cells. This regeneration of *LGR5* + CSCs following ablation of *LGR5 + cells* was also observed in patient-derived organoids.^165^ Additionally, CSCs may enter a state of quiescence in response to therapy, allowing them to persist and tolerate the treatment and increases the risks of tumor recurrence.^166^ Despite these limitations, we are witnessing the emergence of promising epigenetic-based therapeutic strategies for cancer patients, which offer great potential in the years ahead.

**Table 1.** Non-exhaustive list of epigenetic drugs in the treatment of CRC (Adapted from Luo et al.)

Target	Chromatin modification	Drugs	Clinical trial
DNMT1	DNA methylation	5-aza-2ʹ-deoxycytidine (Decitabine)	NCT00879385
			NCT00529022
		5-Azacytidine (5-AzaC)	NCT01193517
			NCT01105377
		Hydralazine	NCT00404508
			NCT00996060
		Procaine	—
		*N*-Phthaloyl-l-tryptophan 1	—
		SGI-1027	—
		(−)Epigallocatechin-3-*O*-gallate	NCT02891538
			NCT01606124
			NCT02321969
			NCT01239095
		Disulfiram	—
		MG98	—
DNMT3A		CBC12	—
		MC3353	—
DNMTs		5-Fluoro-2ʹ-deoxycytidine	NCT01534598
			NCT00359606
		2-Pyrimidone-1-β-d-riboside (Zebularine)	—
		Guadecitabine	NCT03576963
SMYD3	H3K4me3	BCI-121	—
G9a	H3K27me	BIX-01294	—
	H3K9me	UNC0638	—
KDM4C	H3K9me3	FLLL-32	—
LSD1	H3K4me	MC2584	—
	H3K9me	Tranylcypromine	—
EZH2	H3K27me3	Tazemetostat (EPZ-6438)	NCT01897571
			NCT03874455
			NCT02875548
			NCT02601950
		3-Deazaneplanocin A (DZNep)	—
		GSK343	—
		UNC1999	—
		UNC6852	—
I HDAC	Histone acetylation	Valproic acid	NCT04310176
		Romidepsin	NCT00077337
			NCT02512172
		4SC-202	—
I, II HDAC		Resminostat	NCT01277406
		Belinostat	NCT00413322
			NCT00413075
		Trichostatin A (TSA)	—
IIb HDAC		Pivaloyloxymethyl butyrate	—
pan-HDAC		Panobinostat	NCT00690677
			NCT02890069
			NCT01238965
		Sodium phenylbutyrate	NCT00002796
HDAC1-3		Entinostat	NCT02437136
			NCT01105377
			NCT03215264
HDAC2		Vorinostat	NCT00336141
			NCT02316340
			NCT00942266
			NCT00138177
			NCT00126451
			NCT01023737
BRD4		GSK525762 (I-BET762)	NCT01943851
			NCT04116359
			NCT03925428
		OTX-015	NCT02303782
			NCT02259114
		CPI-0610	NCT02157636
			NCT01949883
		NHWD-870	NCT06073938
		JQ-1	—
		BMS-986158	NCT02419417
			NCT03936465
			NCT04817001
			NCT05372354
		PLX51107	NCT04022785
			NCT02683395
			NCT04910152

## Future directions

In this review, we have discussed some of the known epigenetic changes during intestinal stem cell differentiation and how these mechanisms are deregulated in CRC. However, the exact roles of most epigenetic mechanisms in the maintenance and differentiation of ISCs remain poorly understood. For example, the deposition of H2AK119ub downstream of various PRC1 subcomplexes and its interplay with other epigenetic regulators, such as PRC2 and DNA methylation, remain largely elusive. This knowledge is also lacking in CRC development, which is associated with phenotypic reprogramming and cell plasticity. There is a significant gap in our understanding of epigenetic reprogramming in CRC initiation, progression, and therapy resistance. Characterizing these epigenetic changes can offer exploitable targets for diagnosis, prognosis, and therapy management. Advances in this field have been primarily hindered by costly and technically challenging epigenetic profiling techniques. However, recent technological advances have paved the way for more accessible and high-throughput epigenetic profiling approaches that can be applied at low-cell or even single-cell resolution. Furthermore, due to intra-tumor heterogeneity, the epigenetic landscape of different tumor subpopulations (eg, cells located at the tumor invasive front) remains largely unknown. In this regard, the epigenetic landscape of CSCs has yet to be dissected to gain a comprehensive understanding of how colorectal cancer evolves and progresses. These insights will provide more efficient strategies for targeting CSCs to prevent tumor progression or therapy resistance.^167^ For instance, cancer-initiating cells exhibit distinct DNA methylation patterns compared to differentiated tumor cells.^168,169^ Recent research has shown that the heterogeneity in DNA methylation profiles can potentially predict the extent of tumor immune cell infiltration, serving as an indicator of whether immunotherapy could benefit patients.^170^ Another promising approach is inducing the cellular differentiation of CSCs, which may offer a novel strategy to enhance therapy outcomes. However, the development and approval of epigenetic compounds have been limited, emphasizing the urgent need for a thorough evaluation of existing epigenetic drugs for targeting CSCs.

Lastly, recent developments in organoids, which serve as avatars of corresponding tumors, will also help bridge this knowledge gap in the near future.^169,171-173^ Novel engineered models can mimic the environment of an organ in cell culture by controlling the biochemical surroundings of organoids through a fabricated microfluidic system connected to the organoid.^174^ This “organoid-on-a-chip” can be derived directly from organoids, cell lines, epithelial, or stem cells, offering a way to modulate metabolites and chemical gradients in a physiological-like structure.^175,176^ Examples of applications for mini-guts with a microvasculature include studying the gut microbiota and anaerobic bacterial species or the role of the microenvironment in intestinal development or differentiation.^177,178^ The benefits of these intestines-on-chip have been covered in multiple recent reviews.^176,179,180^ This exciting progress promises new ways to explore the epigenetic landscape of CRC within a 3D spatiotemporal model.

## Data Availability

No new data were generated or analyzed in support of this research.
